# “Maxillary lateral incisor partial anodontia sequence”: a clinical entity with epigenetic origin

**DOI:** 10.1590/2177-6709.22.6.028-034.oin

**Published:** 2017

**Authors:** Alberto Consolaro, Maurício Almeida Cardoso, Renata Bianco Consolaro

**Affiliations:** 1 Universidade de São Paulo, Faculdade de Odontologia de Bauru (Bauru/SP, Brazil).; 2 Universidade de São Paulo, Faculdade de Odontologia de Ribeirão Preto, Programa de Pós-graduação em Odontopediatria (Ribeirão Preto/SP, Brazil).; 3 Faculdade de Medicina e Odontologia São Leopoldo Mandic, Disciplina de Ortodontia (Campinas/SP, Brazil).; 4 Faculdades Integradas de Adamantina, Disciplina de Patologia Bucal (Adamantina/SP, Brazil).

**Keywords:** Partial anodontia, Conical teeth, Impacted canines, Unerupted teeth

## Abstract

The relationship between maxillary lateral incisor anodontia and the palatal displacement of unerupted maxillary canines cannot be considered as a multiple tooth abnormality with defined genetic etiology in order to be regarded as a “syndrome”. Neither were the involved genes identified and located in the human genome, nor was it presumed on which chromosome the responsible gene would be located. The palatal maxillary canine displacement in cases of partial anodontia of the maxillary lateral incisor is potentially associated with environmental changes caused by its absence in its place of formation and eruption, which would characterize an epigenetic etiology. The lack of the maxillary lateral incisor in the canine region means removing one of the reference guides for the eruptive trajectory of the maxillary canine, which would therefore, not erupt and /or impact on the palate. Consequently, and in sequence, it would lead to malocclusion, maxillary atresia, transposition, prolonged retention of the deciduous canine and resorption in the neighboring teeth. Thus, we can say that we are dealing with a set of anomalies and multiple sequential changes known as sequential development anomalies or, simply, sequence. Once the epigenetics and sequential condition is accepted for this clinical picture, it could be called “Maxillary Lateral Incisor Partial Anodontia Sequence.”

## RESUMO

A relação entre a anodontia parcial do incisivo lateral e o deslocamento palatino do canino superior não irrompido não pode ser considerada uma anomalia dentária múltipla com etiopatogenia genética definida, a ponto de ser considerada como uma “síndrome”. Os genes envolvidos sequer foram identificados e localizados no genoma humano, e nem mesmo presumiu-se em qual cromossomo se localizaria o gene responsável. O deslocamento palatino do canino superior em casos de anodontia parcial do incisivo lateral superior está potencialmente associado às mudanças ambientais provocadas pela sua ausência no local de formação e erupção, o que caracterizaria uma etiologia epigenética para essa associação. A falta do incisivo lateral superior na região canina implica em tirar um dos guias referenciais da trajetória eruptiva do canino superior, que ficaria, assim, não irrompido e/ou impactado no palato. Como consequência, e em sequência, promove-se uma má oclusão, atresia maxilar, transposição, retenção prolongada do canino decíduo e reabsorções nos dentes vizinhos. Dessa forma, pode-se afirmar que estamos frente a um conjunto de anomalias e alterações múltiplas sequenciais conhecido como anomalias de desenvolvimento sequencial ou, simplesmente, sequência. Uma vez aceita a condição epigenética e sequencial para esse quadro clínico, ele poderia ser chamado de “Sequência da Anodontia Parcial do Incisivo Lateral Superior”.

Partial anodontia, known as hypodontia by Europeans, is a tooth disorder characterized by the absence of one or more teeth and has hereditary etiology. It is not terminologically appropriate to call partial anodontia as congenital, once “congenital” is a quality of what appeared or was possible to be diagnosed at birth. Partial anodontia, especially of permanent teeth, manifests itself only later, with the establishment of dental germs and the formation of odontogenic tissues, not at birth.

Partial anodontia is part of the tooth agenesis group, along with total anodontia. In fact, these terms, although used as synonyms in some situations, are not. The most affected tooth by partial anodontia varies with ethnicity. In Europeans and their descendants, the most evolved teeth are the mandibular and maxillary premolars; in the Japanese, the mandibular central incisors; and in the United States, the maxillary lateral incisors. In Brazil, the prevalence varies with the migratory predominance in each region.^8^ ^,^ ^9^

The biggest prevalence of partial anodontia are the third molars either the four or only one of them, in all ethnic groups, with variations between 25 and 35% in frequency. In addition to its high prevalence, partial third molar anodontia is associated with a great morphological variety as to their position and number. This apparent instability often leads to the non-inclusion of third molar partial anodontia in the various studies on the subject: it would be very unpredictable and could affect the results.

Partial anodontia is hereditary in nature, and the most associated genes are MSX1, PAX9, and AXIN2.^10^ ^,^ ^20^ ^,^ ^25^ ^,^ ^40^ ^,^ ^41^ In the Mendelian pattern of heredity, family cases are mainly autosomal dominant; but there are the recessive ones and even those related to the sex chromosome.^13^ ^-^ ^18^

**Figure 1:**
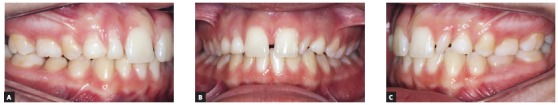
White 13.7 years old patient, Class II (1/4) on the right side and Class I on the left side, with the maxillary middle line slightly shifted to the left. Retention is noted in teeth # 52, # 53, # 55, # 63, # 65 and # 75 with morphological simplification of tooth # 22 of conical shape, and diastema between teeth # 11 and # 21. There was family report of partial anodontia in the maternal grandmother; parents and brother had no partial anodontia.

**Figure 2:**
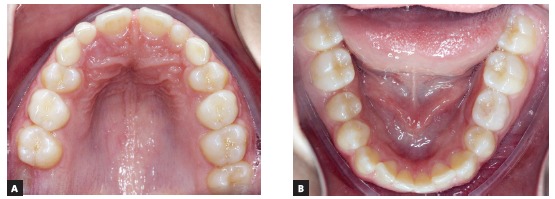
The maxillary deciduous canines did not present mobility and the permanent successors did not present palpable areas in the palatal region. The second molars had already erupted, except for tooth # 17.

**Figure 3:**
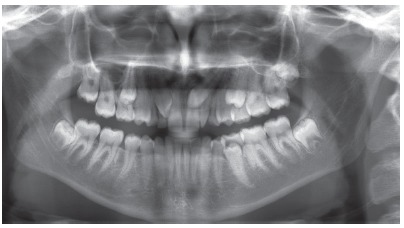
In the panoramic radiograph, the bad positioning of the teeth #13 and # 23 stands out, with absence of the teeth # 12 and # 18. Note the retention of teeth # 52, # 53, # 55, # 63, # 65 and # 75. The permanent maxillary canines were mesially angulated. Tooth # 13 was in a more critical situation, erupting in the position of tooth # 12 and in close contact with the root of tooth # 11. Tooth # 23 was erupting towards the midline, superimposed by the roots of teeth # 21 and # 22.

**Figure 4:**
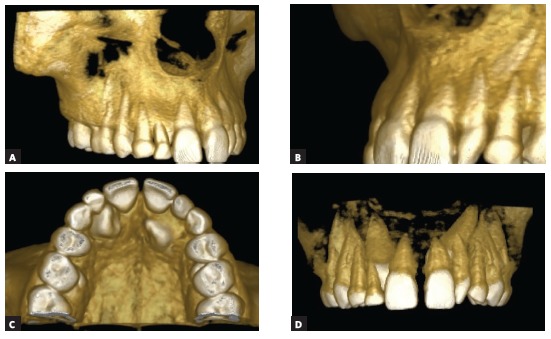
Three-dimensional tomographic reconstructions, with greater precision regarding the spatial location of unerupted canines in the maxillary bone and their relationships with neighboring permanent teeth and the retained deciduous teeth in the dental arch. The conical shape affects crown and tooth root in most of the affected teeth.

The gene associated with partial anodontia has an incomplete penetrance and varied expressivity. When the partial anodontia gene acts, it can lead to the complete absence of the tooth, but may also to its microdontia, with or without its associated conical shape (Fig 1 to 4). Conical teeth as a manifestation of the anodontic gene also affects the shape of roots, leaving them triangular, tapered, and shorter.^42^ The tendency to conical teeth explains the more convergent crowns and simpler or less complex occlusal surfaces. It can be said that microdontic conical teeth are incomplete manifestations of the partial anodontia gene, due to its incomplete penetrance and variable expression.

In patients with partial anodontia, the existing teeth present the phenomenon of morphological simplification,^8^ ^,^ ^22^ ^,^ ^26^ ^,^ ^37^ characterized by:

» reduction of the mesiodistal distance of their crowns;
» lower cusps in a less complex occlusal surface;
» absence or sharp reduction of the cingulum;
» absence or sharp decrease of cups of Carabelli;
» second molars with only three cusps;
» more convergent crowns; and
» shorter and conical roots, compared to normal ones.

## Partial anodontia and unerupted palatally displaced canines and transposition: historical and interesting findings

The impaction of canines was observed in prehistoric,^19^ medieval^39^ skulls, and a few centuries’ old skulls.^7^ The current prevalence varies from 1 to 3% of cases reported^4^. Newcomb^24^ (1959) reported through clinical observations, without any experimentation, that there was a relationship between the canine shifted to the palatal area, dentition development and other anomalies.

The pioneers in establishing a direct relationship between maxillary partial anodontia and palatal maxillary canines impaction were Miller in 1963; followed by Bass in 1967; and Weise in 1969.^1^ ^,^ ^23^ ^,^ ^43^ Only in 1981, Becker et al^2^ ^-^ ^5^ drew new attention and reported the possibility of lateral incisors reduced in size being associated with unerupted palatal canines. Similar results were obtained by Brin et al^6^ in 1986 on the analysis of 2,440 students (1,173 boys and 1,267 girls) aged between 14 and 18 years; further concluding that patients from families with palatally displaced canines were more likely to have the same palatally displaced maxillary canines, small, conical or absent maxillary laterals and late dentition development.

In the 1990s, several authors have published studies relating^11^ ^,^ ^21^ ^,^ ^27^ ^-^ ^36^ ^,^ ^38^ partial anodontia and varied forms of anodontic gene expression with unerupted canines displaced palatally and also with transposition, especially between maxillary and first premolars. These authors have sought to establish this gene relationship, but there is little published work^29^ superficially mentioning external factors, polygenic and multifactorial aspects, without mentioning details or explanations of such phenomena. In 2016, Garib et al^12^ reported an increased risk of children with dental anomalies diagnosed during mixed dentition to develop palatally displaced canines, when compared to children without these anomalies.

## Three theories or mechanisms that would explain the relationship between partial anodontia of the maxillary lateral incisors, unerupted maxillary canines and other alterations

In tooth formation there is not yet an accurate explanation of the mechanisms and genes that determine their morphology, color, number, size and position, and much less of the genes and mechanisms associated with their eruption. Many papers published over the last 70 years have shown that partial anodontia involves a series of dental morphological changes, which is called morphological simplification. Three phenomena or theories (referred to as hypotheses, thoughts, philosophies, theses, schools, currents, etc.) explain why when the number of teeth is modified, morphology, size and other teeth characteristics also change.

These three theories or phenomena, when quoted in articles, are not explained in detail from a clinical point of view, although researchers are also clinicians, and this has caused confusion. In order to be clearly understood, three fundamental concepts, described below, must be explained very well. 

Our body has 25,000 filed and biochemically available data in the nucleus of each of our 10 trillion cells. This set of information is known as genome. In the past, it was thought that our morphology was determined by this set of genes or data received from our parents.

### _^*1st concept and theory: Epigenetics*^_

Genes probably account for about 50% of our characteristics. The other half is determined by environmental factors, that is, where we live and our internal tissue environment, which is where our cells live and develop.

The internal or external environmental factors strongly influence gene functioning without mutating them, which would configure a mutation. When mutations occur, developmental disorders arise in their most varied forms and levels of complexity.

Environmental factors account for approximately half of our final characteristics, including teeth and dentitions. The science that studies environmental factors is known as Epigenetics. Such factors are metabolism, hormones, nutrition, temperature, forces and many others. In teeth and dentition, we have all these epigenetic factors acting until its final formation, and this includes growth forces or vectors, occlusion, addictions, use of braces, etc. The number, color, shape, position, size and time of tooth formation are therefore result of gene action and epigenetic factors.

Explaining unerupted teeth, partial anodontia or its position based only on information provided by genes is to ignore the great importance of epigenetic factors, as important as genetics in the formation and functioning of our body (50 / 50).

The absence of the lateral incisor and its change in shape and size might be epigenetic factors involved in the various forms of canine displacement to the palatal region. Tooth transposition probably also has a strong epigenetic connotation, and this may be one of the aspects that explain some the studies associating it with partial anodontia and unerupted canines. It is hard to imagine human dentition without the influence of epigenetic factors.

**Figure 5:**
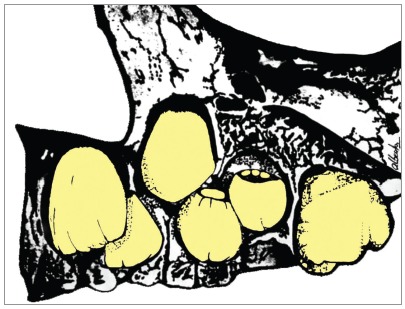
The maxillary lateral incisor is surrounded anteriorly by the central incisor; later, by the canine and first premolar. The canine tends to be located buccally to the lateral incisor; and the first premolar, palatally. The absence of the maxillary lateral incisor, or its diminished size causes the canine to lose one of its references in the eruptive trajectory, even moving to the palatal region.

The space where adjacent teeth to the maxillary canine are formed may be one of the key epigenetic factors. Two aspects should be noted:

An important factor, almost completely disregarded in the lack of space and displacement of the maxillary canines, relates to the 3D space the canine occupies in the same region where the central incisors, laterals and first premolars are. This “maxillary canine region” space is larger or smaller, and more or less malleable, according to the mineralization time of these teeth: of the four, the laterals are the ones that take the most time in mineralization from the crown to the root, as shown in Figure 5. Without the laterals, the canines lose one of the delimiters of this space, as if it were a guide, and they “get lost” in the eruptive trajectory, and might be located palatally. Lateral incisors may be one of the eruption guides for maxillary canines.

2) In the canine region, we have cores of differentiated bone formation, as the growth center of the premaxilla, alveolar process and palatine process. The absence of the maxillary lateral incisor affects the resultant of the forces generated by the growth centers, being able to change these resultants, sometimes referred to as growth vectors. These changes, coupled with the previously mentioned factors can greatly influence the position of the palatally displaced canine, which would primarily be much more associated with epigenetic factors than genetic ones.

**Figure 6:**
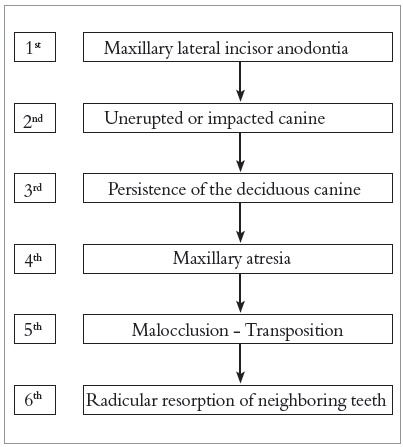
Sequence of anomalies and changes that may be initially induced by anodontia of the lateral incisor, and can be labeled “Maxillary Lateral Incisive Partial Anodontia Sequence.”

Similarly, the position of the maxillary canine may facilitate the sequential occurrence of transposition, rotation, resorption of adjacent teeth, as well as maxillary atresia and malocclusion (Fig 6).

### _^*2nd concept and theory: Pleiotropic Gene, or Pleiotropy*^_

Pleiotropy is the characteristic of some genes functioning in our body, and the gene associated with the human dentition may be one of them. A pleiotropic gene is one that influences or determines various phenotypic characteristics at the same time.

Pleiotropy derives from the Greek pleion (“more numerous”) and tropos (“affinity”). Together they form the word pleiotropic, which represents the phenomenon in which a pair of alleles determines the occurrence of various characteristics in the same organism.

In other words, only one gene can determine the various characteristics of human dentition, such as number, shape, size, color, and position. By changing this gene, various features of the teeth would change, thus justifying the morphological simplification presented in anodontic patients. Among these, there would also be the palatal impaction of canines and transposition.

### _^*3rd concept and theory: “Polygenic System”*^_

The concept of “polygenic system” implies the organization of several smaller genes, acting in a synchronized way, being responsible for several characteristics. By altering one of these genes, such as the one responsible for the number of teeth, other characteristics such as shape, size and even position, would also change. The relationship between partial anodontia and dental position changes only based on clinical and epidemiological findings may be explained as follows, based on the polygenic system: altering the gene responsible for number may alter the adjacent gene responsible for position and time of eruption.

## On multifactorial heredity and partial anodontia versus maxillary unerupted canines

When explaining the palatal impaction of maxillary canines, it is fundamental to know the meaning of the term “multifactorial”. When a multifactorial nature is attributed to an illness, it implies that for it to exist or to happen, the causes must act synergistically, simultaneously, one potentiating or causing the other to cause the disease in question. When a disease has many causes, it is not multifactorial due to this; it only has several causes.

By using the term “multifactorial in heredity”, one must have greater precision on the concept of what is a multifactorial heredity. The most suitable examples of diseases transmitted by parents by multifactorial inheritance are diabetes mellitus and cleft lip and palate.

In multifactorial heredity, the gene has been transmitted from parents to children, but it will only work actively producing the disease, if in association with an environmental or epigenetic factor. In the case of diabetes mellitus, the gene will only induce the transmitted disease if there is obesity, sedentary lifestyle and other epigenetic factors. In the case of cleft lip and palate, the gene will lead to cleft formation if there are environmental factors during pregnancy, such as stress, alcoholism, drugs, nutritional disorders and others. If there is no such association, the disease will not manifest itself.

There is no evidence, however small, that palatal canine impaction is of multifactorial hereditary nature, and it may even be demonstrated in future works, but it is not in the face of the present knowledge.

## The names of multiple anomalies

Multiple anomalies may characterize repetitive clinical pictures in patients and thus receive names and concepts widely used in scientific communication.

**» Syndrome:** it is a set of multiple anomalies related to each other and produced by the action of a single genetic cause, without presenting a sequence of cascade effects. In other words, a syndrome is a clinical picture of multiple anomalies and developmental changes induced by a single cause. As an example, we have the Gorlin and Goltz syndrome, consisting of multiple maxillary keratocysts, nevoid basal cell carcinomas on the skin, and skeletal anomalies induced by the mutation of a tumor suppressor gene on human chromosome 9 (9q22).
**» Association:** is the set of changes or multiple anomalies of a repetitive occurrence in many people, non-occasionally and non-identifiable as a syndrome because it has no known genetic cause. Often, there is an association from which the gene defect is eventually identified, thus failing to be an association and becoming a syndrome. It can also be called syntropy.
**» Sequence:** are multiple defects induced by a sequence of events initially promoted by a known or presumed cause. The sequence may also be called sequential developmental anomaly, as in the Pierre Robin Sequence, which causes the lack of upward movement of the cephalic portion of the fetus. Without this movement, the jaw is prevented from growing because it is caught by the cardiac prominence. Without the full mandibular growth, the tongue does not come down and does not develop normally, remaining between the two secondary palatal processes, preventing the formation of the palate or causing it to assume a high arched form, causing severe occlusal problems.

## Considerations and final concept

The relationship between the partial anodontia of the lateral incisor and the palatal displacement of the unerupted maxillary canine, as well as other possible anomalies of position and shape cannot be regarded as a set of anomalies and changes of defined genetic etiology as to be considered a “syndrome”.

There is clinically an “association” between lateral incisor anodontia, palatal displacement of maxillary canines and other anomalies and disorders (Figs 1 to 4) without the involved genes being identified and located in the human genome - its gene was not even presumed.

The palatal displacement of maxillary canines in cases of partial anodontia of the maxillary lateral incisor is potentially associated with environmental changes caused by its absence in the place of formation and eruption, which characterizes an epigenetic etiology for this association (Fig 5).

The lack of the maxillary lateral incisor in the canine region may mean taking one of the anatomical “guides” of reference for the eruptive trajectory of the maxillary canine. Without this point of reference, the canine would not erupt and remain in intraosseous position, impacted on the palate. Or, it may erupt outside the normal arch, displaced to the palatal region (Figs 1 to 4).

As a result and in sequence, it would promote malocclusion, maxillary atresia, transposition, prolonged retention of the deciduous canine and resorption in neighboring teeth. In this way, one can say that we are facing a set of multiple anomalies and multiple sequential changes known as sequence developmental anomalies or simply sequence (Fig 6). Once the epigenetics and sequential condition for this clinical picture is accepted, it could be called “Maxillary Lateral Incisive Partial Anodontia Sequence” as a new clinical entity to be identified in orthodontic planning.
